# Atypical Presentation of Zieve Syndrome: A Case Report and Literature Review

**DOI:** 10.7759/cureus.85772

**Published:** 2025-06-11

**Authors:** Olaniyi Fadeyi, Helai Hussaini, Saviz Saghari, Nii Tetteh

**Affiliations:** 1 Hospital Medicine, El Centro Regional Medical Center, El Centro, USA; 2 Internal Medicine, West Anaheim Medical Center, Anaheim, USA

**Keywords:** alcohol-related liver disease, hyperlipidemia, jaundice cholestatic, non-immune hemolytic anemia, zieve syndrome

## Abstract

Zieve syndrome is a rare pathology usually diagnosed in patients with a history of alcohol-related liver disease. It is characterized by a triad of Coombs-negative hemolytic anemia, cholestatic jaundice, and hyperlipidemia. Patients usually present with abdominal pain, nausea, jaundice, and ill-defined symptoms. While Zieve syndrome is associated with alcohol-induced liver injury, its mechanism of action is still poorly understood. Nevertheless, most patients respond well to alcohol abstinence, blood transfusions, and supportive care. Herein, we report the case of a 33-year-old female patient who presented to the ED with complaints of abdominal discomfort, intermittent melanotic stools, and hematemesis. Gastroenterology and Oncology were consulted for further evaluation and management. The patient was subsequently diagnosed with Zieve syndrome. In this report, we review this unique case and discuss different atypical forms of presentation in Zieve syndrome, as highlighted in previous studies.

## Introduction

Zieve syndrome (ZS) was first established in 1958 as a triad of non-immune hemolytic anemia, cholestatic jaundice, and hyperlipidemia in the context of alcohol abuse and liver disease. These patients typically present with abdominal pain, nausea, jaundice, and other nonspecific symptoms [1]. Studies have shown that ZS is often under-reported and misdiagnosed [2]. Documented incidence of ZS is around one in 1600 admissions in general wards based on literature [3]. While the exact pathogenesis remains elusive, some hypotheses which may explain the development of ZS have been suggested [4-6]. Generally, most patients improve with alcohol abstinence, blood transfusions, and supportive care. Herein, we present the case of a 33-year-old female patient who was diagnosed with an atypical form of ZS. In addition, various atypical ways of presentation in ZS, as previously documented in literature, are highlighted in this study.

## Case presentation

A 33-year-old female patient with a past medical history of morbid obesity, methamphetamine abuse, and chronic alcohol abuse with resultant liver cirrhosis presented to the ED with complaints of abdominal discomfort, intermittent melanotic stools, and hematemesis. She denied use of non-steroidal anti-inflammatory drugs (NSAIDs) and blood thinners. Initial vital signs were normal. Physical exam revealed lower abdominal tenderness, jaundice, and scleral icterus. Labs revealed severe anemia, thrombocytopenia, neutropenia, coagulopathy, transaminitis, hyperbilirubinemia, and hyperammonemia. Lipid panel was unremarkable. Other notable laboratory studies on admission are summarized in Table 1.

**Table 1:** Initial laboratory results with the respective reference ranges WBC: white blood count; PT: prothrombin time; INR: international normalized ratio; PTT: partial thromboplastin time; HCO_3_: bicarbonate; BUN: blood urea nitrogen; AST: aspartate aminotransferase; ALT: alanine transaminase; LDH: lactate dehydrogenase; LDL: low-density lipoprotein *Significant laboratory results

Laboratory parameters	Results	Reference range
WBC* (X 10^3^/µL)	2.86	4.8-10.8
Hemoglobin* (g/dL)	5.9	13.7-17.5
Hematocrit* (%)	17.4	38.8-50
Platelets* (X 10^3^/µL)	22	150 -450
PT* (seconds)	38.1	11-13.5
INR*	3.3	0.8-1.1
PTT* (seconds)	50	25-35
Sodium (mmol/L)	138	136-145
Potassium* (mmol/L)	2.8	3.5-5.3
Magnesium* (mg/dL)	1.4	1.7-2.2
HCO_3_ (mmol/L)	24	22-26
BUN (mg/dL)	3	6-24
Creatinine (mg/dL)	0.49	0.6-1.2
Blood glucose (mg/dL)	109	70-140
Lipase (U/L)	104	0-160
AST* (U/L)	120	8-48
ALT (U/L)	25	7-55
Alkaline phosphatase* (IU/L)	160	30-147
Total bilirubin (mg/dL)	20.7	0.2-1.2
Direct bilirubin (mg/dL)	10.7	0.0-0.3
Indirect bilirubin (mg/dL)	10	0.2-0.8
Albumin* (g/dL)	2.6	3.6-5.1
Ammonia* (µmol/L)	179	11-32
LDH* (U/L)	411	40-280
Haptoglobin* (mg/dL)	<10	30-200
Reticulocyte percent* (%)	8.88	0.5-2.5
Total cholesterol (mg/dL)	102	<200
Triglycerides (mg/dL)	40	<150
LDL (mg/dL)	70	<100

Abdominal ultrasound result was significant for bidirectional flow in the main portal vein, which is typical of liver cirrhosis. The patient was admitted into ICU for upper gastrointestinal bleed and transfused with multiple units of packed red blood cells (pRBC). She was started on sucralfate, protonix drip, and octreotide drip. She received fresh frozen plasma (FFP) and prothrombin complex concentrate with an obvious improvement in the international normalized ratio (INR). The patient was also placed on rifaximin, lactulose, vitamin K, spironolactone, furosemide, and ceftriaxone for spontaneous bacterial peritonitis prophylaxis. Her model for end-stage liver disease (MELD) score was 34. Acute hepatitis panel was unremarkable. Coagulation parameters improved with treatment as shown in Table 2.

**Table 2:** Results of the liver function tests and the coagulation panel during hospital admission ALP: Alkaline phosphatase; AST: aspartate aminotransferase; ALT: alanine transaminase; PT: prothrombin time; INR: international normalized ratio

Component	Reference range	Day 1	Day 2	Day 3	Day 4	Day 5	Day 6	Day 7	Day 8
Albumin (g/dL)	3.5-5.2	2.6	2.5	3.2	3	3.2	2.7	2.5	2.7
Total protein (g/dL)	6.6-8.7	7.3	6.9	8.1	7.7	8.1	7.4	6.9	7.2
Total bilirubin (mg/dL)	0.0-1.2	20.7	20.6	26.2	23.7	22.4	17.8	16	16.3
ALP (U/L)	40-129	160	122	128	119	120	133	158	139
AST (U/L)	0.0-41	120	101	101	87	90	73	67	62
ALT (U/L)	5-40	25	24	27	26	26	21	19	19
PT (seconds)	11-13.5	38.1	28.8	24.1	24.7	25.6	25.2	24.8	24.5
INR	0.8-1.1	3.3	2.5	2.1	2.2	2.7	2.6	2.5	2.2

**Figure 1:**
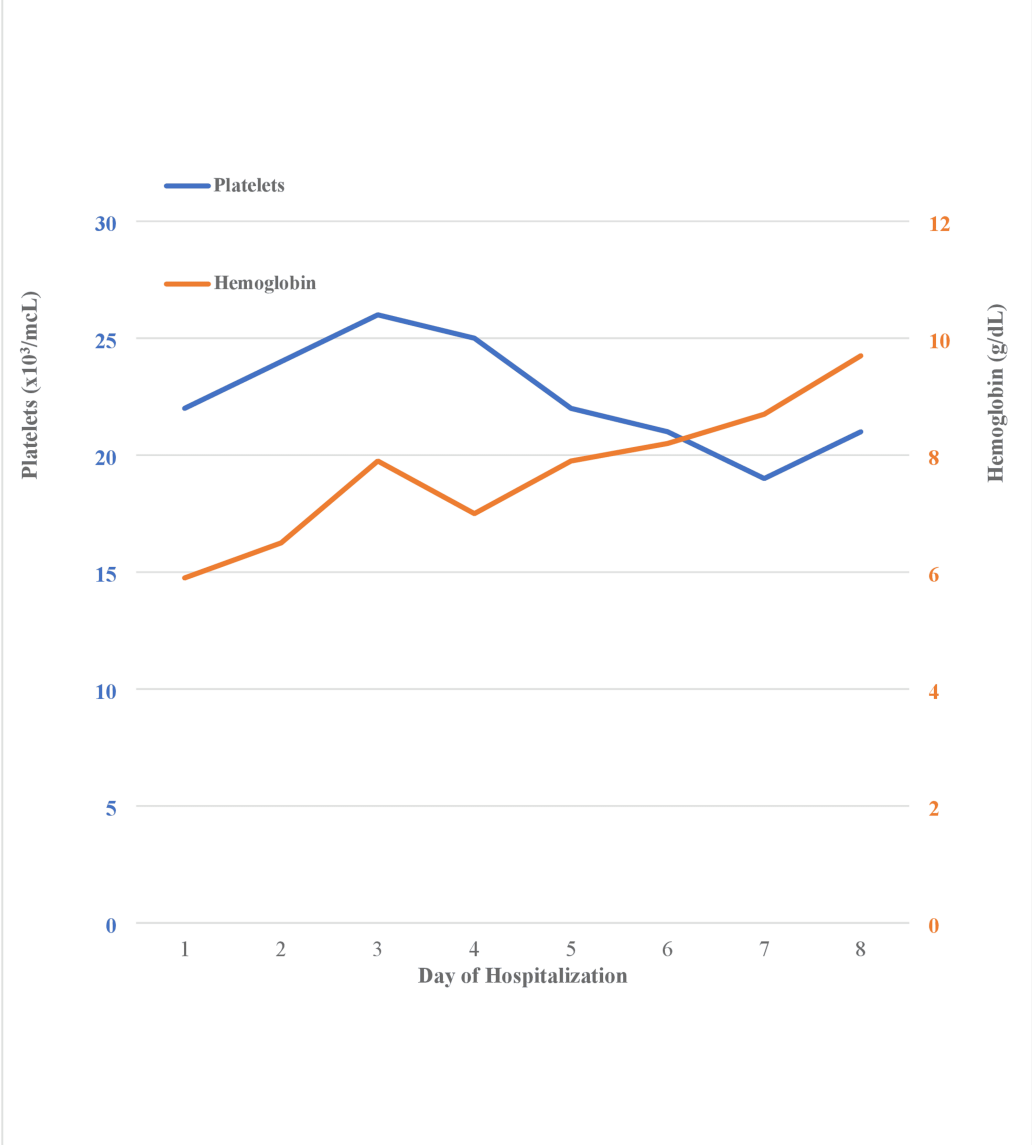
Complete trend of hemoglobin and platelet levels during the hospital admission

Esophagogastroduodenoscopy (EGD) procedure performed by the gastroenterologist revealed portal hypertensive gastropathy without oozing and unremarkable foregut examination without stigmata of hemorrhage or esophageal varices. Hemoglobin level responded mildly to multiple units of pRBC transfusion, while the patient remained thrombocytopenic throughout the admission despite receiving 8 units of platelets (Figure 1).

Further work-up by Hematology-Oncology was consistent with non-immune hemolytic anemia. Direct Coombs test was negative. Our patient was eventually diagnosed with ZS associated with liver failure. She was discharged in a stable condition and referred for a liver transplant.

## Discussion

ZS is an underreported clinical manifestation seen in patients with a history of alcohol abuse. Typically, this disease presents with a triad of Coombs-negative hemolytic anemia, cholestatic jaundice, and hyperlipidemia. While the pathophysiology of ZS remains obscure in mainstream literature, some hypotheses have been proposed to explain the occurrence of hyperlipidemia, hemolytic anemia, and jaundice. For instance, it was suggested that the dysregulation of hepatic lipases in a damaged liver results in fat mobilization, thereby triggering transient hyperlipidemia in ZS [4]. Hemolysis, on the other hand, is believed to be triggered by two different mechanisms. First, it is precipitated by high levels of lysolecithin and lysocephalin, which are released due to hepatocellular damage caused by hyperlipidemia [5]. Second, alcohol-induced vitamin E deficiency triggers a decline in erythrocyte glutathione and polyunsaturated fatty acids, which weakens the RBC plasma membrane and eventually results in hemolysis [6]. Jaundice seen in ZS is a direct consequence of hyperbilirubinemia caused by a combination of hemolysis and hepatocyte injury triggered by alcohol-induced intrahepatic cholestasis [4].

Meanwhile, more cases of atypical presentations of ZS have been seen in clinical settings. For instance, our patient presented with hemolytic anemia and jaundice without any form of hyperlipidemia. This is consistent with previous studies, which placed the incidence of hyperlipidemia in ZS at around 50% [1]. Hyperlipidemia is easily missed because it fluctuates and normalizes one to two weeks following an acute episode [7]. While hyperlipidemia may be part of the triad necessary to diagnose ZS, its absence (as seen in our patient) does not totally rule out the diagnosis.

Other forms of atypical presentation of ZS have been documented in literature. For example, Choudhry et al. [8] reported a case of severe hypertriglyceridemia in a 46-year-old patient who was diagnosed with ZS. This patient initially presented to the ED with complaints of right upper quadrant abdominal pain. Physical examination was significant for generalized jaundice of the skin. Lipid panel revealed hypertriglyceridemia at 4,425 mg/dL (normal reference value: <150 mg/dL) and elevated total cholesterol at 872 mg/dL (normal reference value: <200 mg/dL). Plasmapheresis was completed to correct hypertriglyceridemia. The patient was discharged home on statin, fenofibrate, and omega-3-acid ethyl esters. He was counselled to stop alcohol. Subsequent follow-up with the primary care physician (PCP) was uneventful.

Furthermore, Vedire et al. [9] described a case of a unique variant of ZS in a 44-year-old female patient. Detailed work-up revealed hyperlipidemia and hemolytic anemia with a normal reticulocyte count. The patient initially presented with complaints of weakness and lethargy. Physical examination was significant for mild abdominal distention, scleral icterus, and jaundice. Meanwhile, high reticulocyte count is always associated with hemolytic anemia. However, hemolytic anemia with a normal reticulocyte count in this context was probably due to weakness in the bone marrow suppression triggered by chronic alcohol abuse, which eventually precipitated an inadequate response in the hemolytic environment. Nevertheless, the patient responded well to alcohol cessation and a short course of prednisolone.

It is imperative for clinicians to be aware of atypical presentations in ZS to avoid unnecessary diagnostic interventions and invasive biliary procedures [10]. This is because a presentation of right upper quadrant abdominal pain and jaundice could easily mimic choledocholithiasis and ascending cholangitis. Recommended treatment for ZS remains alcohol abstinence and blood transfusions as needed [11]. Most patients typically recover within four to six weeks. Additionally, use of plasmapheresis has been found to be very helpful in patients presenting with hypertriglyceridemia. While not typically recommended in every case, high risk patients with a history of pancreatitis and intracerebral hemorrhage have been found to benefit from plasmapheresis to foreclose the possibility of complications arising from hypertriglyceridemia [12]. Meanwhile, use of steroids in ZS has not been found to be helpful. Corticosteroids use is helpful in autoimmune hemolytic anemia, which is not consistent with the variant of hemolytic anemia found in ZS [13]. Use of steroids in ZS could trigger increased morbidity and mortality along with an increased incidence of infection [4]. 

Persistent thrombocytopenia seen in our patient was probably due to her history of liver cirrhosis. Nevertheless, the patient was discharged in a stable condition. She was asked to abstain from alcohol and follow up with her PCP in preparation for a liver transplant.

## Conclusions

ZS may have unique presentations, but Coombs-negative hemolytic anemia is typical. Regardless of the different forms of presentation, abstinence from alcohol, blood transfusions, and supportive care remain the standard of care. Physicians who come across acute hemolytic anemia in a patient with a history of alcohol abuse and liver disease should consider ZS as a differential diagnosis. Further, it is important for clinicians to maintain a high index of suspicion when assessing patients presenting with right upper abdominal pain and jaundice to avoid unwarranted diagnostic and therapeutic interventions, which may have a very low yield in the context of the standard treatment for ZS.
